# Validation of the Early Language Scale

**DOI:** 10.1007/s00431-020-03702-8

**Published:** 2020-06-13

**Authors:** Margot I. Visser-Bochane, Cees P. van der Schans, Wim P. Krijnen, Sijmen A. Reijneveld, Margreet R. Luinge

**Affiliations:** 1 Research Group Healthy Ageing, Allied Health Care and Nursing, Hanze University Groningen, University of Applied Sciences, Petrus Driessenstraat 3, 9714 CA Groningen, The Netherlands; 2 Department of Rehabilitation Medicine, University Medical Center Groningen, University of Groningen, Groningen, The Netherlands; 3 Department of Health Psychology Research, University Medical Center Groningen, University of Groningen, Groningen, The Netherlands; 4 Department of Health Sciences, University Medical Center Groningen, University of Groningen, Groningen, The Netherlands; 5 Department of Otorhinolaryngology, Head & Neck Surgery, University Medical Center Groningen, University of Groningen, Groningen, The Netherlands

**Keywords:** Screening, Developmental language disorder (DLD), Prevention, Child health surveillance, Language development

## Abstract

The aim of this study was to assess the criterion validity of a new screening instrument, the Early Language Scale (ELS), for the identification of young children at risk for developmental language disorder (DLD), and to determine optimal age-adjusted cut-off scores. We recruited a community-based sample of 265 children aged 1 to 6 years of age. Parents of these children responded on the ELS, a 26-item “yes-no” questionnaire. The children were assessed with extended language tests (language comprehension, word production, sentence production, communication). A composite score out of these tests (two tests below – 1 SD or one below − 1.5 SD) was used as reference standard. We assessed the validity of the ELS, measured by sensitivity, specificity, predictive values, and AUC. The optimal sensitivity/specificity age-dependent cut-off ELS score was at 15th percentile. Sensitivity and specificity were 0.62 and 0.93, respectively. Positive predictive value was moderate (0.53), negative predictive value was high (0.95), the positive likelihood ratio was 9.16, and negative likelihood ratio was 0.41. The area under the ROC curve was 0.88. The items covered the increasing language development for the ages from 1 to 6.

*Conclusion*: The ELS is a valid instrument to identify children with DLD covering an age range of 1 to 6 years in community-based settings.

**Table:** 

**What is Known:** *• Early identification and treatment of developmental language disorders can reduce negative effects on children’s emotional functioning, academic success, and social relationships.* *• Short, validated language screening instruments that cover the full age range of early childhood language development lack.* **What is New:** *• The 26-item Early Language Scale (ELS) is a valid instrument to identify children at risk for developmental language disorder in well-child care and early educational settings among Dutch children aged 1–6 years.*

**What is Known:**

*• Early identification and treatment of developmental language disorders can reduce negative effects on children’s emotional functioning, academic success, and social relationships.*

*• Short, validated language screening instruments that cover the full age range of early childhood language development lack.*

**What is New:**

*• The 26-item Early Language Scale (ELS) is a valid instrument to identify children at risk for developmental language disorder in well-child care and early educational settings among Dutch children aged 1–6 years.*

## Introduction

Developmental language disorder (DLD) is one of the most common developmental problems in children and has a negative effect on children’s emotional functioning, academic success, and social relationships [1–5]. Its estimated prevalence is 7% [6]. Early identification and treatment of DLD can prevent or reduce its detrimental effects [7], and preventive child health services may offer an excellent setting for early identification.

Early identification of DLD in well-child care requires short, valid, and reliable instruments, that preferably cover the age range in which language develops. A widely used short instrument for delineated age categories in a large age range is the Ages and Stages Questionnaire (ASQ) [8]. The ASQ is a simple parental questionnaire to identify children with suspected developmental delays in communication, gross motor, fine motor, problem solving, and the personal-social domain. However, instruments that target only language development outperform the ASQ communication domain in identifying atypical language development [9, 10]. These instruments for language screening, also listed in the US Preventive Task Force (USPSTF) update [9, 11, 12], are the MacArthur-Bates Communicative Development Inventory (CDI) [13] and Language Development Survey (LDS) [14]. Both instruments are too lengthy for routine use in well-child care as they require parents to check at least 100 and 310 items, respectively. Recently, CDI short forms were introduced, comprising only 25 items, confirming the need for shorter instruments that focus on language development. However, this CDI short form is only suitable for children up to 30 months of age [15], whereas the moment of identification of DLD typically exceeds this age [16–20]. Currently, there is no valid instrument available that covers the full early developmental period in which language is developed, i.e., up to 6 years, and that is also short to administer.

We developed an Early Language Scale (ELS)—that measures language development in 26 easy observable items by parents, covering the full range of early language development (1–6 years). However, evidence on its validity and scoring is lacking. The aim of this study was therefore to assess the criterion validity of the ELS, and to determine optimal age-adjusted cut-off scores.

## Methods

### Study design

This study was the validation part of a cross-sectional study on the development of an Early Language Screening instrument (ELS) for the measurement of language development in children (registered at trialregister.nl: 5746). In the current study, we compare the results of the ELS with a reference standard in a community-based cohort of children aged 1 to 6 years. This study was approved by the Medical Ethical Committee of the University Medical Center of Groningen (M13.134252 / NL45253.042.13).

### Sampling and participants

**Fig. 1:**
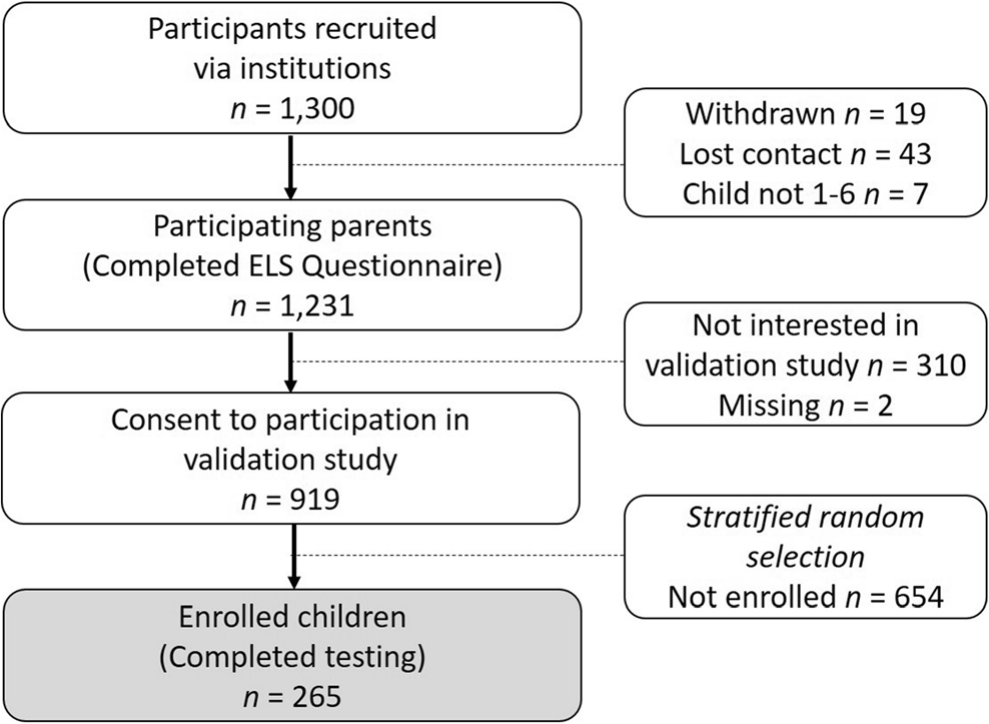
Flowchart of participant recruitment

We recruited parents and their children from 1 to 6 years of age in a two-step procedure. In the first step, we recruited well-child clinics, kindergartens, and schools. Second, these institutions recruited parents by distributing our folder, and collecting and returning reply forms. We asked them to do so according to predefined guidelines on the sample required (e.g., parents of the five youngest 3-year-old boys), in order to create a well-balanced sample regarding age and sex distribution. Exclusion criteria were significant sensory impairments (e.g., blindness, deafness), or mental disorders (e.g., mental retardation). In total, 1231 parents of children from 1 to 6 were recruited via 162 institutions and provided normative data. In this study, we validated the Early Language Scale (ELS) in a subsample of the normative sample. For this subsample, all participating parents were invited to let their child participate in the validation part of the study (Fig. 1). In total, 919 (74%) parents agreed. Out of these, a random sample for validation regarding children with Dutch as their first language was invited for testing, stratified by gender and age year of the child. All parents provided written informed consent. Characteristics of the sample are presented in Table 1. The sample was representative for the Dutch population with respect to birthweight, and pregnancy duration. Participating children were more likely to have mothers who were more highly educated. For comparison, 56% of the females (25–35 years old) have a higher education level, vs 68% in our sample (Statistics Netherlands, 2019).

**Table 1:** Characteristics of the sample by age category

	15–23 months	24–35 months	36–47 months	48–59 months	60–72 months	Total
	(*n* = 35)	(*n* = 58)	(*n* = 59)	(*n* = 58)	(*n* = 55)	(*n* = 265)
Characteristics	*n* (%)	*n* (%)	*n* (%)	*n* (%)	*n* (%)	*n* (%)
Gender						
Male	16 (46%)	26 (45%)	35 (59%)	29 (50%)	25 (45%)	131 (49%)
Female	19 (54%)	32 (55%)	24 (41%)	29 (50%)	30 (55%)	134 (51%)
Birthweight (grams)*						
High (> 5000)	-	-	-	-	-	-
Average (2500–5000)	35 (100%)	55 (95%)	54 (92%)	51 (88%)	52 (95%)	247 (93%)
Low (< 2500)	-	3 (5%)	2 (3%)	6 (10%)	2 (4%)	13 (5%)
Pregnancy duration						
Serotine (> = 42 weeks)	5 (14%)	3 (5%)	3 (5%)	1 (2%)	9 (16%)	21 (8%)
Term (37–42 weeks)	30 (86%)	52 (90%)	53 (90%)	48 (83%)	44 (80%)	227 (86%)
Preterm (< 37 weeks)	-	3 (5%)	3 (5%)	9 (16%)	2 (4%)	17 (6%)
Language						
Dutch only	31 (89%)	52 (90%)	52 (90%)	50 (86%)	49 (89%)	235 (89%)
Dutch and other language(s)	4 (11%)	6 (10%)	6 (10%)	8 (14%)	6 (11%)	30 (11%)
Maternal education*						
High	28 (80%)	41 (71%)	41 (70%)	36 (62%)	35 (64%)	181 (68%)
Middle	7 (20%)	14 (24%)	16 (27%)	20 (35%)	18 (33%)	75 (28%)
Low	-	3 (5%)	2 (3%)	1 (2%)	2 (4%)	8 (3%)
Maternal age*						
21–30	11 (31%)	20 (35%)	23 (39%)	27 (47%)	26 (47%)	107 (40%)
31–35	16 (46%)	27 (47%)	28 (48%)	20 (35%)	20 (36%)	111 (42%)
> 36	7 (20%)	7 (12%)	8 (14%)	11 (19%)	6 (11%)	39 (15%)

*Numbers do not always add up to *n* = 265 due to missing data, birthweight: 5 missing, maternal education: 1 missing, maternal age: 8 missing

### Procedure

Data for validation were collected by speech-language pathologists from March 2015 to July 2016 during a home visit of, on average, 2 h. During the home visit, we first obtained informed consent. Thereafter, we collected from the parent the following: the filled-in parent forms belonging to the reference language tests, background characteristics of the child and the family, and responses to the ELS questionnaire. During the collection of these data, the children could get used to the speech-language pathologist. Lastly, we administered reference tests to the children, in order to provide a reference standard for the validation of the ELS. Children of 2 years of age and older were assessed with specific language tests on language comprehension, followed by language production. Children younger than 2 years of age are too young for these tests. Therefore, we assessed their language comprehension and language production with parental questionnaires. Finally, we assessed communication with a standardized observational instrument for children up to 4 years of age. Communication of children aged 4 and 5 was assessed with a standardized parental questionnaire. The tests used are described below.

### Measurements

#### Early Language Scale

The Early Language Scale (ELS) is a parental questionnaire consisting of 26 yes/no questions on the language development of children in the age of 1 to 6 years old (Appendix 1). The items were administered in Dutch. We used forward-only translation of the original items to English for international publication. A yes score results in one point; the ELS total score represents the sum of all items a child has acquired, this score varies from 0 to 26. The ELS consists of items on language development, within the domains of vocabulary (semantics), sentences/grammar (syntax and morphology), and communication (language use). In a prior study, using the data from a community-based sample (*n* = 1231), items were selected from an item bank of 75 items based on the criteria of Mokken scaling, which is based on the nonparametric Item Response Theory [21]. Items were identified via automated item selection procedure [22], evaluated on monotonicity and item ordering, resulting in a final ELS scale consisting of 26 items. The constructed ELS was a strong scale (scalability coefficient *H* = 0.83) with Item *H* coefficients between 0.62 and 0.90 [23]. This shows that the scale consists of the most distinctive items that describe the development of language in children aged one to six years.

#### Reference tests

For the validation of the ELS, we assessed the language development of the child with age-appropriate reference tests of language development, i.e., the core concept to be assessed by the ELS. The ELS encompasses items regarding language comprehension and production (of vocabulary and grammar), and communication. The reference tests measured the same aspects of language development; therefore, the reference standard is a combination of language tests that assess the aspects language comprehension and production (of words and sentences), and communication. Table 2 gives an overview of tests used per age category. We constructed a reference standard as the composite score out of these tests, calculated as follows: two or more test scores below − 1 SD of the norm score or 1 test score (on language comprehension or production) below − 1.5 SD of the norm score resulted in a deviant language score. We denoted children with a deviant language score as children with atypical language development; this includes DLD and language delays. The tests used are described below.

**Table 2:** Tests used per language domain and age year

Age (months)	Language comprehension	Language production		Language use/communication
12–23				
	Lexilist Comprehension (LLC)	Lexilist Production (LLP)		
				Language Standard – Communication Composite Score (LS-CCS)
24–35	Schlichting test for Language Comprehension (SLC)	Schlichting test for Word Production (SWP)	Schlichting test for Sentence Production (SSP)	
36–47				
48–59				CCC-2-NL- Pragmatic Composite Score (CCC-PCS)
60–72				

The *Lexilist Comprehension (LLC)* [24] and *Lexilist Production (LLP)* [25] to measure language comprehension and language production in children younger than 2 years of age comprise lists of words and phrases (190 words and 25 phrases for comprehension, and 263 words and 11 phrases for production) for parents to tick the words and phrases of which they think their child understands, and uses. Results vary from 0 to 225 (LLC), and 0 to 274 (LLP), and an age-standardized score (mean = 100; SD = 15) were calculated according to the manuals [24, 25]. Internal consistency of the LLC is good (Cronbach’s alpha of .98). The LLP shows good reliability and sufficient validity [26].

The *Schlichting tests for Language Comprehension (SLC)* [27], *Word Production (SWP)* [28], and *Sentence Production (SSP)* [28] to measure language comprehension and language production in children from 2 to 7 years of age. The SLC is a 85-item test assessing comprehension of grammatical constructions using toys, pictures, and tokens. The SWP is a 70-item test assessing expressive vocabulary using a stimulus booklet with pictures. The SSP is a 40-item test assessing expressive grammatical constructions, using imitation of expressions, visualized in a stimulus booklet with in some cases associated toys. Age-standardized scores for each test (mean = 100; SD = 15) were calculated according to the manuals, in which also entry levels per age, and cut-off rules are described [27, 28]. The SLC, SWP, and SSP have excellent internal consistency (lambda-2 = .93, .93, and .90, respectively) [27, 28].

The *Language Standard (LS)* [29] is a 20-item observational instrument, providing information on general language ability. The trained professionals observed the child while playing with the parent and with the professional on standardized observation items, and scored findings on a 5-point scale according to the manual “5 points: clear evidence for normal; 4 points: between 5 and 3; 3 points: possible evidence for problem; 2 points: between 3 and 1; 1 point: clear evidence for problem.” [29]. We extracted a composite score on communication (LS-CCS), based on consensus of six professionals, including the authors MVB and ML. Consensus on items that regard communication was reached in two consensus rounds prior to testing. In the first round, all professionals indicated which items from the Language Standard regarded communication. In the second round, professionals received their own scores and the scores of all other professionals. They were then asked to indicate which items regarded communication again. This resulted in consensus on 8 items, regarding the observation of the child’s communication skills while playing with the parent. Observed items were, for example, initiation of contact, responds in communication, attention for language, nonverbal communication. Counting the scores on these items (5, 6, 7, 8, 12, 17, 18, and 20) results in a maximum score of 40, with a cut-off at 32 or lower indicating a problem on communication.

The *Children’s Communication Checklist – 2 – NL (CCC)* [30] is a 70-item parental checklist that assesses children’s communication behaviors over 10 subscales. The sum of the scales E–H (E. inappropriate initiation, F. stereotyped language, G. use of context, H. nonverbal communication) provides a composite score on pragmatics (CCC-PCS). This frequently reported score identifies children likely to have significant pragmatic language problems. Psychometric properties for the Dutch adaptation of the CCC-2 are satisfactory; with internal consistency ranging from .53 to .75 [30]. The subscale pragmatics shows sufficient reliability, but insufficient criterion validity [31].

#### Background characteristics

We obtained data by parent report on the following background characteristics: age of the child, gender, birthweight, length of pregnancy, language situation at home, highest level of education achieved by the mother, maternal age at birth of the child, and family history (sibling, parent, grandparent) of language delay*.* Education level was classified into three categories: low (primary school or less, and pre-vocational education), middle (secondary education), and high (higher vocational education and university).

### Analyses

First, we selected children with two or more reference test scores − 1 SD or more below the mean or one reference test score (on language comprehension or production) − 1.5 SD or more below the mean in order to identify these children as children with atypical language development. Second, we assessed the criterion validity of the ELS. To visualize our data, we plotted the ELS total score against age, and added smoothed splines, representing group means, one for the children with typical language development and one for children with atypical language development, based on the combined reference standard. Next, we calculated sensitivity and specificity indices, positive predictive value and negative predictive value, and positive likelihood ratio and negative likelihood ratio, for various age-adjusted cut-offs of the ELS against the reference standard. The age-adjusted cut-off values of the ELS were calculated for 3 months age groups regarding 5th, 10th, 12th, 15th, 25th, 50th, 75th, and 100th percentiles within our norm sample (*n* = 1231). We chose 3 months age groups to be able to differentiate within an age year, as children achieve several language milestones per age year. We also performed ROC analyses determining the area under the curve (AUC), by calculating sensitivity and specificity for the various cut-off values. Finally, we assessed whether the performance of the ELS differed by background characteristics.

## Results

### Sample

All children spoke Dutch as first language. Atypical language development, based on the combined reference standard, was found in 29/265 (11%) of the children. These 29 children were from all ages (five 1-year olds; nine 2-year olds; eight 3-year olds; four 4-year olds; three 5-year olds). One child spoke Dutch as first language and another language at home, one child had a birthweight below 2500 g, five children were born at a pregnancy duration of less than 37 weeks, and two children had mothers with a low education level.

### Outcomes on the Early Language Scale and the reference standard

**Fig. 2:**
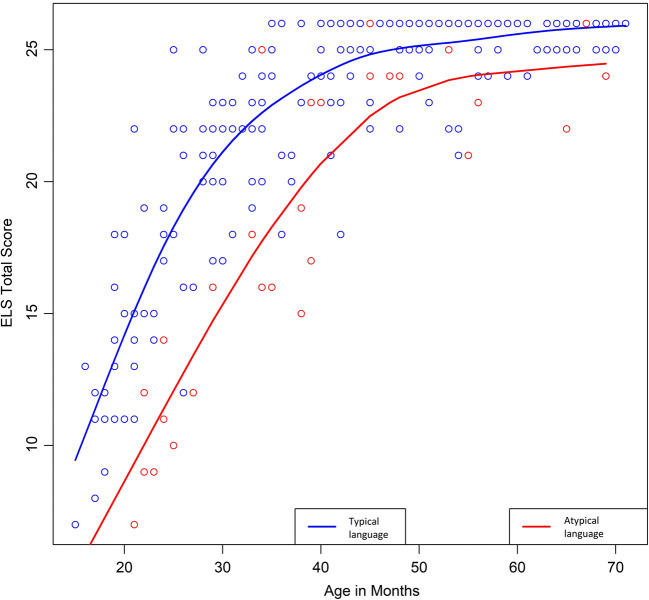
Early Language Scale total scores by age in months, for children with typical language development in blue (*n* = 236) and children with atypical language development in red (*n* = 29). Lines refer to group means. The median scores on the ELS test were 11 for 1 year old, 20 for 2 years old, 24 for 3 years old, 25 for 4 years old, and 26 for 5 years old

ELS total scores for children with a typical language development, based on the reference standard, were higher than for children with an atypical language development. ELS total scores for both groups increased across ages, with a ceiling effect at the maximum score of 26 on the ELS (Fig. 2).

### Validity

**Fig. 3:**
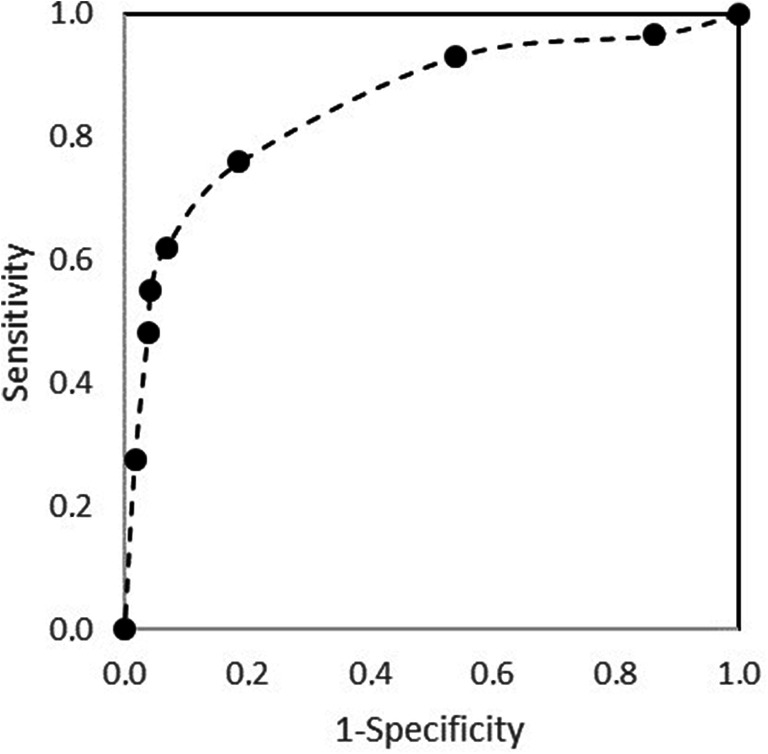
Receiver operating curve regarding the ability of the Early Language Scale (ELS) to distinguish children with atypical language development and children with typical language development

The receiver operating curve (ROC) was built using various age-adjusted cut-offs of the ELS. The area under the receiver operating curve (AUC) in Fig. 3 was 0.88, indicating that the ELS adequately differentiated children with atypical from those with typical language development. Taking a cut-off at the 15th percentile, to provide an optimal balance between sensitivity and specificity while minimizing over-referral rates, resulted in a sensitivity of .62, a specificity of .93, a positive predictive value of .53, a negative predictive value of .95, a positive likelihood ratio of 9.16, and negative likelihood ratio of .41 (Table 3). This regarded the following cut-off values per age: 12–14 months, 2; 15–17 months, 6; 18–20 months, 7; 21–23 months, 9; 24–26 months, 14; 27–29 months, 15; 30–32 months, 16; 33–35 months, 18; 36–38 months, 20; 39–41 months, 21; 42–44 months, 22; 45–53 months, 23; 54–71 months, 24. We applied the cut-off values as follows: for a child of 23 months old, the cut-off value of 9 means endorsement of a maximum of 9 items results in a deviant score, endorsement of 10 or more items results in a normal score.

**Table 3:** Results of the screening instrument (ELS) and the reference standard

Result reference standard	Identified by screening *n* = 34	Not identified by screening *n* = 231	Total *n* = 265
Atypical language	18	11	29
Typical language	16	220	236

### Differences in the performance of the ELS by background characteristics

Finally, we assessed whether the performance of the ELS differed by background characteristics, i.e., for the four subgroups: true positives, false positives, false negatives, and true negatives of the ELS. We found no significant effect of birthweight (*F*(3, 256) = 1.19, *p* = 0.315), Length pregnancy (*F*(3, 261) = 2.47, *p* = 0.06), maternal age (*F*(3, 253) = 1.32, *p* = 0.27) assessed using one-way ANOVA. There was also no significant effect of gender (*X*^2^ (3, *N* = 265) = 0.69, *p* = 0.88). Maternal education and language could not be tested as these had too little observations in one or more subgroups. Maternal education low is more prevalent in the true positive group, confirming that children from low educated mothers have slower language development. Regarding language situation at home, most of the children with Dutch and another language spoken at home had typical language development and were not identified by the screening, indicating that the language situation did not affect the performance of the ELS. Finally, results were similar if the 17 included preterm children were excluded (*p* = 0.248, Fisher’s exact test). In general, results confirmed expectations.

## Discussion

This study examined the validity of the Early Language Scale (ELS) in children aged 1 to 6 using a composite score of language tests as reference standard. We found that the ELS has a good validity making use of only a limited set of items feasible for an age range of 1 to 6 years.

We found that the ELS has a good validity, i.e., an AUC of .88, comparable with the CDI and LDS with reported AUC’s of .86 and .85, respectively [32, 33]. However, in contrast to these instruments, the ELS covers a large age range (1 to 6); the AUC holds for the entire age range in which the basics for language develops, meaning that the ELS measures this developmental ability. Moreover, the ELS is a valid instrument to measure language over the developmental period of 1 to 6 years of age.

We found a sensitivity of .62 and a specificity of .93, which is suitable for community-based screening. A high specificity, of at least .90, minimizes over-referrals with its negative effects, e.g., discomfort for parents and children, and costs [34]. On the other hand, the sensitivity of the ELS is moderate, but increasing sensitivity by larger cut-off values would decrease specificity. A higher sensitivity is desirable, however not at the expense of lower specificity. This contrasts with screening of high-risk populations in which the balance between sensitivity and specificity should shift towards a higher sensitivity, to minimize under referrals with its negative effects, e.g., progressive language problems and associated long-term effects. The ELS is thus suitable for community-based screening of children in a rather large age range.

The properties of the ELS, e.g., a limited set of 26 items reflecting a broad definition of language development that is also feasible for a large age range, make the ELS an addition to other valid language screening instruments. The ELS exceeds the age range of the MacArthur-Bates Communicative Development Inventory (CDI) short forms [15] and the Language Development Survey (LDS) [14]. Both CDI and LDS regard vocabulary checklists filled by parents. However, LDS assesses only expressive language and not receptive language. The ELS included items on expressive and receptive language. Atypical language development in children aged 1 to 6 are easy to detect with only a small number of items; however, some children will still be missed.

The scale has been developed in Dutch, but consists of items that are very common in related languages, such as English and German. For instance, an item like “says two-word sentences” (item 11) is rather similar in those languages. Use of the scale in other languages requires additional research to obtain language-specific norms and a validation in that language.

A major strength of our study is that we used a large, community-based sample, equally distributed over gender, and age groups of 1 to 6 years, with state of the art reference tests. We tested the power of our sample based on the assumption of a prevalence of 11% atypical language development and 89% typical language development (according to the reference standard) in our sample, i.e., the prevalence under the independence. Both resulting *p* values were < 0.001, indicating that the results of the ELS are not based on coincidence, and therefore, the sample of *n* = 265 was sufficient to assess validity. However, a limitation of this study is that despite its size, the number of cases was still relatively small. This limited the potential to analyze outcomes per age year. Another limitation of our study is that the sample had some overrepresentation of mothers with a high education level. This resulted in cut-off norms that might be slightly low for children from disadvantaged families, as evidence suggests that children of highly educated mothers (and fathers) have better language [35]. However, the norms are feasible for our sample, including the full range of educational levels. Moreover, a better language development is associated with successful developmental and educational outcomes. Therefore, all children with atypical development are entitled to identification of this crucial deficit. Lastly, children develop language with individual variability and speed. Therefore, it is recommended to monitor language development over time. The ELS regards ordered language items and can be used repeatedly.

The results of this study show that the ELS is a valid instrument for the detection of children at risk for developmental language disorder in a population-based sample. The ELS can provide support to professionals working in well-child care and early educational settings to quickly identify children (1–6) with an atypical language development. This identification of children supports timely referral to proper diagnostics and intervention. This may improve these children’s emotional functioning, academic success, and social relationships.

The validity of the 26-item ELS is satisfactory for a broad age range. Further research is recommended to investigate its age-specific validity and long-term predictive value, as well as the applicability of its set-up to other languages. The use of the ELS in routine community settings deserves further study regarding feasibility and effects in a clinical setting.

## Conclusion

The ELS is a valid instrument to identify children at risk for DLD covering an age range of 1 to 6 years in community-based settings. The ELS is promising for identifying children with atypical language development in a public health setting as it does not yield a high proportion of false positives.

## Appendix. The Early Language Scale (ELS)

**Table:**

Name child: Age child (in months):			
	ELS item	Yes	No
1	Does your child say any ‘words’? For example: ‘*mama*’, ‘*papa*’, ‘*cookie*’? It doesn't have to be pronounced correctly.		
2	When you play with your child, with a ball for example, does your child have attention for you and the ball?		
3	Does your child understand tasks consisting of two words? For example: ‘*coat on*’ or ‘*look there*’.		
4	Does your child understand when you ask him/her something? For example: ‘*Shall we read a book together?*’		
5	Does your child understand 3 word sentences? For example: ‘*on the chair*’ or ‘*to the hallway*’.		
6	Can your child point out something you ask them to? For example: ‘*where is your nose?*’ or ‘*where is the ball?*’		
7	Can your child say about ten words in total?		
8	Is your child able to point 5 pictures in a book when they are verbally offered to him/her?		
9	Is your child able to point out 6 body parts on a doll/themselves? *Where are the eyes, mouth, tummy, foot, hair, hand?*		
10	Does your child ask you when he/she wants to have some food, or wants to play with toys?		
11	Is your child able to combine two words? For example: ‘*daddy ball*’ or ‘*look cat*’.		
12	Can your child name four or more pictures of animals? For example: ‘*dog*’, ‘*cat*’, ‘*horse*’, ‘*cow*’.		
13	Is your child able to talk with you in turns?		
14	Is your child able to initiate a conversation?		
15	Are the words in the sentences of your child mostly in the right place?		
16	Does your child ever spontaneously tell you a story? For example, about what they did that day.		
17	Does your child use words that say something about other words (adjectives)? For example, ‘*big*’ in ‘*a big house*’		
18	Is your child able to name a couple of colours correctly?		
19	Can your child retell a story with the help of pictures? For example when reading a book together.		
20	Does your child use words like ‘*we*’, ‘*he*’, and ‘*she*’ in a sentence? For example: ‘*He is really happy*’.		
21	Does your child make sentences with words like ‘when’ or ‘and’? For example: ‘*when we have finished dinner, we are going to play with clay*’ or ‘*they put their coat on and they put their shoes on*’.		
22	Does your child ask questions beginning with ‘*why*’?		
23	Does your child use the proper plural form? For example ‘*feet*’ instead of ‘*foots*’.		
24	Can your child complete the following sentences: *Not black but…. Not high but….*		
25	Does your child make sentences with ‘*because*’?		
26	Does your child use the correct form of the past? For example: ‘*went*’ instead of ‘*goes*’ or ‘*had*’ instead of ‘*haved*’.		
	Total ‘yes’:		

Note: The original items in Dutch are available on request. The authors hold the intellectual property right and they welcome any further use if aligned with them

## Abbreviations

CCC: Children’s Communication Checklist
CDI: MacArthur-Bates Communicative Development Inventory
DLD: Developmental language disorder
ELS: Early Language Scale
LDS: Language Development Survey
LLC: Lexilist Comprehension
LLP: Lexilist Production
LS: Language Standard
SLC: Schlichting test for Language Comprehension
SSP: Schlichting test for Sentence Production
SWP: Schlichting tests for Word Production
